# Role of prespecified analysis plans in physiological research: Encouraged or mandatory?

**DOI:** 10.1113/EP092034

**Published:** 2025-02-21

**Authors:** Robin Christensen, Tobias Haugegaard, Janus C. Jakobsen

**Affiliations:** ^1^ Section for Biostatistics and Evidence‐Based Research the Parker Institute, Bispebjerg and Frederiksberg Hospital Copenhagen Denmark; ^2^ Research Unit of Rheumatology, Department of Clinical Research University of Southern Denmark, Odense University Hospital Odense Denmark; ^3^ Cochrane Denmark & Centre for Evidence‐Based Medicine Odense (CEBMO), Department of Clinical Research University of Southern Denmark Odense Denmark; ^4^ Copenhagen Trial Unit, Centre for Clinical Intervention Research, The Capital Region Copenhagen University Hospital – Rigshospitalet Copenhagen Denmark; ^5^ Department of Regional Health Research, The Faculty of Health Sciences University of Southern Denmark Odense Denmark

A prespecified statistical analysis plan (SAP) is utilized in many research fields to increase the reproducibility of the findings and minimize bias in data analysis and presentation. Extending this prespecified paradigm to experimental physiology research is crucial. Defining methods and analyses in advance allows researchers to increase transparency, allowing reviewers and readers to assess whether the research aligns with its original goals, thereby fostering trust in the findings. Researchers who prespecify their SAP and avoid adjusting their approach based on outcomes also benefit from more objectivity of results. Given that *Experimental Physiology* often publishes studies with exploratory paradigms, we emphasize the benefits of adopting prespecified analysis plans. Such an approach not only improves methodological rigor and transparency but also enhances the quality and reliability of research. Our proposal aligns with the recommendations of the International Committee of Medical Journal Editors (ICMJE), aiming to bolster rigor, reproducibility and the overall quality of research published in *Experimental Physiology*. While *Experimental Physiology* or most physiology journals do not currently mandate prespecified analysis plans, we advocate for their broader adoption to enhance transparency and reproducibility in research, especially in studies that inform clinical practice.

In the context of research or studies, ‘prospective’ often refers to a design or approach where data is collected over time from the present onward rather than relying on past data or retrospective analysis. Prospective research begins after the research question or hypothesis is formulated: researchers design the study, select a study population or cohort, and plan data collection methods before data collection starts. Prospective research designs allow researchers to gather data as events unfold, minimizing recall bias and providing more accurate information about the sequence of events. Prospective research is primarily characterized by a prespecified protocol (Bandholm et al., 2017). Prospective research generally offers greater scientific validity than retrospective research because its predefined methodology lowers the risk of biased, data‐driven results that are not generalizable and may be difficult to replicate. When preparing for an experimental study, it is essential to outline the key aspects of the forthcoming data analysis in the statistical segment of the protocol. This section should encompass the components of the planned main analyses for the outcome variable(s) and outline strategies for addressing potential analysis challenges. In exploratory trials, this section may provide a broader overview of guiding principles and approaches that facilitate innovative research, emphasizing flexibility, iterative learning and the diverse approaches necessary for generating valuable hypotheses (Chan et al., 2013). The exploratory nature of most physiological studies questions the value of prespecifying a physiologically relevant difference or a hierarchy of outcomes if it is not relevant to the aim of the study.

A detailed SAP is a prerequisite for ensuring the validity, reliability and replicability of study findings, as it is essential for upholding methodological rigor. A well‐crafted SAP contributes to the interpretability of research by providing a structured approach to data analysis, but this must be underpinned by a robust study design and high‐quality data collection methods. The purpose of a SAP is to help researchers conduct rigorous and transparent analyses that adhere to best practices and ethical standards in research. It is meant as a comprehensive document that provides a more in‐depth and technical elaboration of the core aspects of the analysis outlined in the protocol (Gamble et al., 2017). It should include detailed procedures for conducting the statistical analysis of both primary and secondary outcome variables, as well as other data of relevance. Ideally, the SAP should be developed (and registered or published) between the finalization of the protocol and before ‘First Patient Last Visit’ (FPLV) or ‘Last Patient First Visit’ (LPFV). However, this terminology may not be applicable in studies involving animal models or where cells serve as the units of analysis. This vague rule of thumb, indicating the timing before data closure, is crucial to ensure the faithful execution of planned analyses and the preservation of data integrity throughout the study. The development and agreement upon the analysis plan usually occur during a blind review with no actual data, which involves the examination of tables and figures with empty cells (commonly referred to as mockups).

Traditionally, mockup figures and tables have been used in SAPs for clinical trials, and they may seem more suited for clinical trials with predefined result presentations. However, we believe they also provide significant benefits in experimental physiology research. The primary purpose of these mockups is to predefine the presentation of results, which helps minimize the risk of rationalizing biased *post hoc* omissions and spin. Additionally, mockups facilitate the communication of design concepts, such as column headings and table structures, and allow for feedback from stakeholders and co‐authors before finalizing the manuscript. By visualizing the design early in the process, mockups help identify potential issues and refine design elements, ensuring that the final presentation is both clear and aligned with anticipated journal requirements. We argue that this approach contributes to a more organized and rigorous reporting of results when the outcomes are ‘unpredictable.’ These mockups explicitly depict the anticipated outline, listing variables, groups, and contrasts between these, usually presented with 95% confidence intervals derived directly from the prespecified objectives (Christensen et al., 2023). While *P*‐values have their utility in hypothesis testing scenarios, confidence intervals offer a more comprehensive and informative summary of the data, aiding in the interpretation and communication of research findings. Confidence intervals provide an estimate of the range within which the ‘true’ population parameter is likely to lie based on the precision of the study. They give a sense of both the magnitude and the precision of the effect being studied, which is more informative than a simple indication of whether the effect is statistically significant (Christensen et al., 2023).

As an overarching principle, authors planning to submit their manuscript to a journal should be familiar with the guidelines available on standard reporting frameworks formulated for various study designs, such as CONSORT for randomized trials, STROBE for observational studies and ARRIVE for animal research (Christensen et al., 2013). Per the recommendations of the ICMJE, proficiently crafted manuscripts present their findings coherently throughout the text, figures and tables, prioritizing the main or most important results at the outset. The primary and key secondary outcomes are usually identified a priori in the study protocol and represent the key measures used to assess the effectiveness or impact of the intervention/exposure being studied. Thus, creativity should be kept to a minimum when the choice is made to present data about all primary and key secondary outcomes outlined in the protocol and described in the methods section. Additional supplementary materials and technical details may be included in supplementary files with appropriate appendices to ensure accessibility without disrupting the text's coherence. Alternatively, they can be exclusively published in the electronic version of the journal. Prospective research typically involves collecting data moving forward, while retrospective analysis relies on historical data, complicating true pre‐specification. This distinction between hypothesis‐testing (prospective) studies and exploratory studies is crucial. Clarifying these differences can significantly enhance the interpretation of study findings and help delineate the objectives and methodologies employed.

## Content of a prespecified analysis plan

Following the current best practice, it is strongly advised to include a flow diagram at the outset of the results section as ‘Figure 1’. These diagrams visually depict the progression of participants throughout each phase of a longitudinal research endeavor, starting from initial recruitment and culminating in the analysis stage, including a clear indication of (the anticipated) missing data issues. In the final manuscript, an exemplary flow diagram enables readers to grasp the progression of participants, encompassing the number screened, allocated to each experimental group, receiving either intervention/exposure or control/comparator, completing the study, and being included in the analysis. It aids in evaluating the study's internal validity by highlighting potential biases like attrition or dropout rates. Moreover, it promotes transparency and simplifies the interpretation and reproduction of the study's results.

After this ‘Figure 1’ has assisted the reader in understanding what comprises the ‘intention‐to‐infer from’ population, baseline measures should be reported by providing a summary of key participant characteristics for each study group in this specific population (i.e., a ‘Table 1’) (Christensen et al., 2023). This encompasses demographic information (such as age, sex/gender, and race/ethnicity), pertinent medical history and other baseline characteristics that could potentially impact the outcomes under investigation. Including all relevant pre‐exposure clinical variables in this table is essential, as these represent the baseline level for the outcomes in subsequent analyses. Descriptive statistics such as means and standard deviations (or medians and interquartile range for apparently non‐normally distributed data) and counts with proportions should be presented for continuous and categorical variables. This comprehensive reporting ensures transparency and allows readers to assess the comparability of the study groups at baseline. In general, displaying baseline data in a table introduced before any intervention (or exposure) effects on outcome variables enhances the clarity, transparency and interpretability of research results, thereby bolstering the robustness and credibility of the research findings. In reference to the descriptive statistics presented in this ‘Table 1’, it is important to note that confidence intervals and standard errors are inferential rather than descriptive. Therefore, they are not suitable for representing baseline characteristics (Moher et al., 2010).

Due to the powerful impact of figures in leaving a lasting impression of results, their creation – and readers' interpretation of them – must be approached with caution (Pocock et al., 2008). A ‘Figure 2’ often includes visual representations of key data or findings relevant to the main research objective, that is, again, the choice should be heavily influenced by what was listed as the primary research question. Such a figure should preferably display a graphical representation of the primary study endpoint and/or treatment effects. The content of this ‘Figure 2’ should be determined by the specific objective(s) and requirements of the research project, as well as the researchers' preferences at the protocol stage.

## Outcomes

Trial results are often better displayed in a table than in the text. When reporting the main analyses from a research project, a table format allows for the presentation of detailed numerical outcome data in a structured manner for both the primary and all key secondary outcomes. This comprehensive presentation gives readers access to specific values and allows for a thorough examination of the data. The presentation of results for both primary and key secondary outcomes should include data for each group, along with the estimated effect size (some contrast between groups) and its precision, typically represented by a 95% confidence interval (95% CI) in a ‘Table 2’. For each outcome listed in this ‘Table 2’, study results should include a summary of the outcome in each group, such as the number of participants with or without the event and their denominators or the (least squares) mean and standard error. Additionally, the contrast between the groups, known as the effect size (or summary measure), should be reported (Guyatt, Thorlund, et al., 2013). For binary outcomes, the summary measure could be – listed in prioritized order – the risk ratio (the same as relative risk), risk difference or odds ratio (Guyatt, Oxman, et al., 2013). For most binary data reporting, both a relative and an absolute measure should be reported (Moher et al., 2010). For survival time data, it could be the hazard ratio or difference in median survival time. Confidence intervals should be provided for the contrast between groups, known to statisticians as the ‘estimand.‘ It is important to avoid the common error of presenting separate confidence intervals for the outcome in each group instead of for the treatment effect (contrast between groups).

The 95% CIs are commonly used, although other confidence levels are occasionally employed. The popularity of the 95% CI is a combination of historical precedent, ease of interpretation, balance between precision and coverage, and practical considerations in research and statistical analysis. This aligns with R. A. Fisher's proposal of the 5% significance level as a conventional threshold for statistical significance. Fisher suggested this level to strike a balance between minimizing Type I errors (false positives) and maximizing the ability to detect genuine effects. Independent of the numerical threshold, confidence intervals offer value when differences between groups fail to reach conventional statistical significance, often indicating the possibility of an important clinical difference (Christensen et al., 2023). Although these thresholds are rarely embraced in studies within experimental physiology, a prespecified minimal important difference can provide a broader foundation for interpreting the results. The utilization of (95%) confidence intervals for communicating ‘null findings’ has seen a significant rise in major journals in recent years, though not uniformly across all medical specialties. Although *P*‐values should still be presented alongside confidence intervals, it is essential not to rely exclusively on *P*‐values for interpreting results. All planned primary and key secondary outcomes should be reported rather than concentrating only on statistically significant findings or those considered ‘interesting’ (Christensen et al., 2023; Williams et al., 2023). Physiological variables are frequently used as surrogate outcomes in clinical trials to offer early insights into intervention effects (Christensen et al., 2024). However, interpreting these variables without knowing the minimal important difference risks overstating their clinical relevance. Changes in physiological measures may not correspond to meaningful improvements in patients (Christensen et al., 2024). Without this threshold, even statistically significant results may lack practical value for clinical decision‐making and patient care, as they may not reflect real‐world benefits (Manyara et al., 2024). Findings should be presented for all planned primary and key secondary outcomes, rather than solely focusing on statistically significant or deemed ‘interesting’ analyses, as a precautionary note, it is considered fraudulent behavior to misconduct and ‘deselect an outcome or test’ after seeing that it failed to reject the null hypothesis. It may be difficult for some experimental physiologists to identify the applicability of standardized outlines for manuscript components to non‐clinical research contexts. However, we believe the standardized outlines could be helpful in the field (see Table 1).

**TABLE 1:** Manuscript outline items and content.

Manuscript outline item	Content (purpose)
(1) Figure 1	Flow diagram illustrating the progression through the phases of a longitudinal study, stratified by groups, including enrolment, intervention allocation, follow‐up and data analysis.
(2) Table 1	Baseline demographic and clinical characteristics reported with stratification by group (representing pre‐exposure levels).
(3) Figure 2	A graphical display of data that communicates the outcome of the primary objective.
(4) Table 2	The primary and key secondary outcomes should be reported as summary results for each study group, with a focus on highlighting the differences between groups, presented alongside (95%) confidence intervals. The inclusion of *P*‐values may be considered optional.

## Perspectives

Structured preregistrations and predefined SAPs are valuable in clinical studies (Bandholm et al., 2017). However, some may argue that these approaches are not always suitable for basic experimental research involving humans, animals and cells (Richter et al., 2024). Experimental physiologists and basic scientists often advocate for a more exploratory approach, emphasizing the value of serendipitous findings – unexpected discoveries that arise when researchers pursue inquiries without rigid constraints. This perspective encourages curiosity‐driven exploration, enabling scientists to follow intriguing observations or anomalies that may not align with predetermined hypotheses. The unpredictable outcomes in basic research further highlight the need for flexibility (Richter et al., 2024). Many physiological mechanisms likely operate in concert, where changes in one variable may have significant effects without directly translating into clinical outcomes; thus, clarifying whether a study's objectives focus on mechanistic insights or clinically relevant outcomes is essential to ensure accurate interpretation and proper distinction between primary and secondary outcomes.

Our advocacy for prespecified plans in basic experimental science should be seen as an attempt to mitigate the broader, well‐documented reproducibility crisis in the life sciences (Macleod et al., 2014). The *Lancet* series on reducing waste and increasing value in biomedical research highlighted that a significant portion of research inefficiency stems from poorly designed studies, selective reporting and lack of transparency (Moher et al., 2016). Many medical journals recognize that prespecified analysis plans address these issues by enhancing methodological rigor, minimizing bias and improving the reliability and replicability of results. By promoting their use, particularly in experimental physiology, we aim to contribute to a culture of transparency and reproducibility that ultimately increases research value and reduces resource waste. Prospective research involves data collection from the present onward, contrasting with retrospective analysis, which relies on past data where true pre‐specification is unlikely. Prospective research starts after hypothesis formulation, allowing for planned study design, participant selection and data collection methods. A prespecified protocol guides prospective research, particularly in experimental trials, where the SAP ensures rigorous and transparent analyses. A successful SAP details statistical procedures for primary and secondary outcome variables and should be finalized before data closure. Mockups (‘empty’ figures and tables) aid in SAP development, reducing the risk of rationalizing and biased data‐driven conclusions and facilitating stakeholder feedback to ensure alignment with research objectives. Manuscripts should adhere to reporting frameworks like CONSORT and STROBE, prioritizing primary and key secondary outcomes in the main text. Flow diagrams enhance transparency, illustrating participant progression and potential biases caused by missing data mechanisms. Baseline characteristics should be detailed in a table for comparability assessment. Outcomes refer to the result or consequence being studied or measured in an experiment: figures effectively communicate primary endpoint findings, while tables present comprehensive outcome data, including effect sizes and 95% confidence intervals for all key secondary outcomes. When studies work from operational objective(s), they also inform the choice of estimand; a well‐defined objective ensures that the estimand accurately captures the desired effect to be measured and interpreted in the study. Fortunately, the use of confidence intervals has increased, providing valuable insights even when results lack statistical significance. *P*‐values should complement, not substitute, confidence intervals, emphasizing all planned endpoints rather than significant findings alone.

While we primarily highlight the importance of prespecified analysis plans in human studies, it is essential to recognize their relevance in animal and laboratory research as well (Percie du Sert et al., 2020). In human studies, these plans help minimize biases and enhance the reproducibility of results, addressing ethical and legal considerations. Similarly, in animal and laboratory research, prespecified plans can guide the iterative process of experimentation, ensuring that studies are well‐defined and transparent (Percie du Sert et al., 2020). Researchers can optimize experimental conditions and better control for confounding variables by clearly outlining methodologies and analytical strategies. Ultimately, adopting prespecified analysis plans across all types of research promotes scientific rigor and facilitates a more reliable comparison of findings, contributing to a more robust understanding of physiological processes (Moher et al., 2016). Recently, Rasmussen et al. published an editorial with the journal emphasizing that preregistration and registered reports enhance research transparency and rigor across exploratory and hypothesis‐driven studies (Rasmussen et al., 2025). We concur that these (novel) practices mitigate bias, reduce publication bias and prevent selective reporting. Registered reports ensure methodological rigor before data collection and guarantee publication regardless of outcomes, fostering transparency and promoting the dissemination of negative and null findings. By integrating these principles, research quality and credibility improve, supporting interdisciplinary collaboration and innovation (Rasmussen et al., 2025). Given the absence of standardized reporting guidelines for mechanistic and exploratory studies, we advocate for developing such frameworks to enhance transparency and improve the quality of research in this area. In contrast to the principles from medical research that we advocate for in experimental physiology, some researchers may argue that registering clinical trials and mandating prespecified analysis plans are valuable only when evaluating clinical outcomes in patient studies, mainly drug trials, to ensure these outcomes remain unaltered (Richter et al., 2024). As a result, we recognize that our proposed guidelines on prespecified analysis plans are unlikely to become mandatory for the journal in the near future, as some stakeholders may find them irrelevant or even counterproductive (Richter et al., 2024).

While this editorial introduces a new paradigm, encouraging the incorporation of prespecified analyses even in exploratory research, we urge the journal to foster an open debate on implementing such rigorous initiatives – already mandatory in most medical journals. Our goal is not to bureaucratize the research process or limit the discovery of novel findings but rather to ensure that the broader implications of research are fully realized while minimizing waste and increasing value.

## AUTHOR CONTRIBUTIONS

Conception or design of the work: Robin Christensen and Janus C. Jakobsen. Acquisition, analysis, or interpretation of data for the work and drafting the work or revising it critically for important intellectual content: all authors. The authors confirm that they have approved the final version of the manuscript and agree to be accountable for all aspects of the work, ensuring that any questions related to the accuracy or integrity of any part of the work are appropriately investigated and resolved. All individuals listed as authors meet the criteria for authorship, and no one who qualifies for authorship has been omitted.

## CONFLICT OF INTEREST

All authors declare that they have no financial conflicts of interest to disclose. However, it should be noted that two of the authors (R.C. and J.C.J.) regularly collaborate as trialists with Contract Research Organizations (CROs), which may be perceived as a potential intellectual conflict of interest.

## FUNDING INFORMATION

The authors gratefully acknowledge the support of the Parker Institute and the Copenhagen Trial Unit in facilitating this research. Section for Biostatistics and Evidence‐Based Research, the Parker Institute, Bispebjerg, and Frederiksberg Hospital is supported by a core grant from the Oak Foundation (OFIL‐24‐074).
